# Unidirectional porous beta-tricalcium phosphate and hydroxyapatite artificial bone: a review of experimental evaluations and clinical applications

**DOI:** 10.1007/s10047-021-01270-8

**Published:** 2021-04-23

**Authors:** Toru Funayama, Hiroshi Noguchi, Hiroshi Kumagai, Kosuke Sato, Tomokazu Yoshioka, Masashi Yamazaki

**Affiliations:** Department of Orthopaedic Surgery, Faculty of Medicine, University of Tsukuba, 1-1-1 Tennodai, Tsukuba, Ibaraki, 3058575 Japan

**Keywords:** Artificial bone, Unidirectional porous structure, Bone grafting, Hydroxyapatite, Beta-tricalcium phosphate

## Abstract

In Japan, where allograft bone transplantation is not widespread, prospects for artificial bones are very high. Therefore, artificial bones with various compositions, porous structures, and porosities have been developed and employed for clinical use. Both Affinos^®^ and Regenos^®^ (made of beta-tricalcium phosphate and hydroxyapatite, respectively) are artificial bones with a unique unidirectional porous structure, in which pores with a diameter suitable for tissue penetration (25–300 μm) are aligned in one direction. The unidirectional porous structure allows rapid penetration of blood deep into the materials by a capillary effect. In animal experiments, Affinos^®^ showed well-balanced resorption and was replaced with the host’s own bone from an early stage after implantation and new bone formation and remodeling were observed in the cortical bone and medullary cavity. When implanted for clinical situation, resorption from an early stage and good replacement with the patient’s own bone were also observed. Regenos^®^ has an internal osteon-like material and a vascular-like structure that is maintained within the pores even after long-term implantation, as noted in an animal experiment. When implanted for clinical situation, good osteoconductivity was observed from an early stage of implantation. In addition, the material was observed to be slowly absorbed over time in some cases. We have discussed the beneficial effects of combining teriparatide and platelet-rich plasma impregnation and the potential prospects of these artificial bones.

**Supplementary Information:**

The online version contains supplementary material available at 10.1007/s10047-021-01270-8.

## Introduction

Bone grafting is frequently performed in orthopedic surgery [1]. The types of grafted bones vary depending on the country, and in Japan, current trends indicate that autologous bones account for 56.4% of grafts, followed by artificial bones at 40%, and allogenic bones at 3.6% [2]; the latter are used in limited cases, such as filling massive bony defects in hip replacements [3]. Although autologous bones are ideal filling materials, there is a risk of complications associated with harvesting [4]. Since the number of patients with osteoporosis is increasing due to the rapidly aging population in recent years, the quantity and quality of autogenous bones that can be harvested are limited. Thus, in Japan, the prospective applications for artificial bones in orthopedic surgery are high. Artificial bones with various compositions, porous structures, and porosities have been developed and employed for clinical use (Table 1). Artificial bones currently used in orthopedic surgery in Japan are mainly composed of beta-tricalcium phosphate (β-TCP), a resorbable bone regeneration material, and hydroxyapatite (HAp), which is not usually replaced by bone.

**Table 1:** Major artificial bone ceramics products clinically available for orthopedic surgery in Japan

Composition	Product name	Manufacturer	Porosity (%)		Pore size (μm)	Pore structure	Interconnection
β-TCP	Affinos	Kuraray		57	25–300	Unidirectional	Y
	Osferion	Olympus Terumo Biomaterial		75	100–400	Spherical	Y
	Osferion 60	Olympus Terumo Biomaterial		60	100–400	Spherical	Y
	Superpore	HOYA Technosurgical		75, 68	50–300	Spherical	Y
	Superpore EX	HOYA Technosurgical		57	40–200	Spherical	Y
	CERAREBONE-H	NGK Spark Plug		35	200	Spherical	Y
	β-Bone	KatalyMedic		75	100–400	Spherical	Y
	β-Bone 60	KatalyMedic		70	100–400	Spherical	Y
HAp	Regenos	Kuraray		75	100–300	Unidirectional	Y
	NEOBONE	CoorsTek		75	150	Spherical	Y
	NEOBONE X	CoorsTek		Dense and 75% complex	150	Spherical	Y
	Apaceram	HOYA Technosurgical		5–60	50–500	Spherical	N
	Apaceram AX	HOYA Technosurgical		85	50–300	Spherical	Y
	Boneceram K	Olympus Terumo Biomaterial		Dense	NA	NA	N
	Boneceram P	Olympus Terumo Biomaterial		35–48	50–300	Spherical	N
β-TCP + HAp	CERATITE	NGK Spark Plug		35% and 50% complex	5,170 complex	Spherical	Y
HAp + Colagen	REFIT	HOYA Technosurgical		95	100–500	Sponge like	Y

*β-TCP* beta-tricalcium phosphate; *HAp* hydroxyapatite; *Y* yes; *N* none

**Fig. 1:**
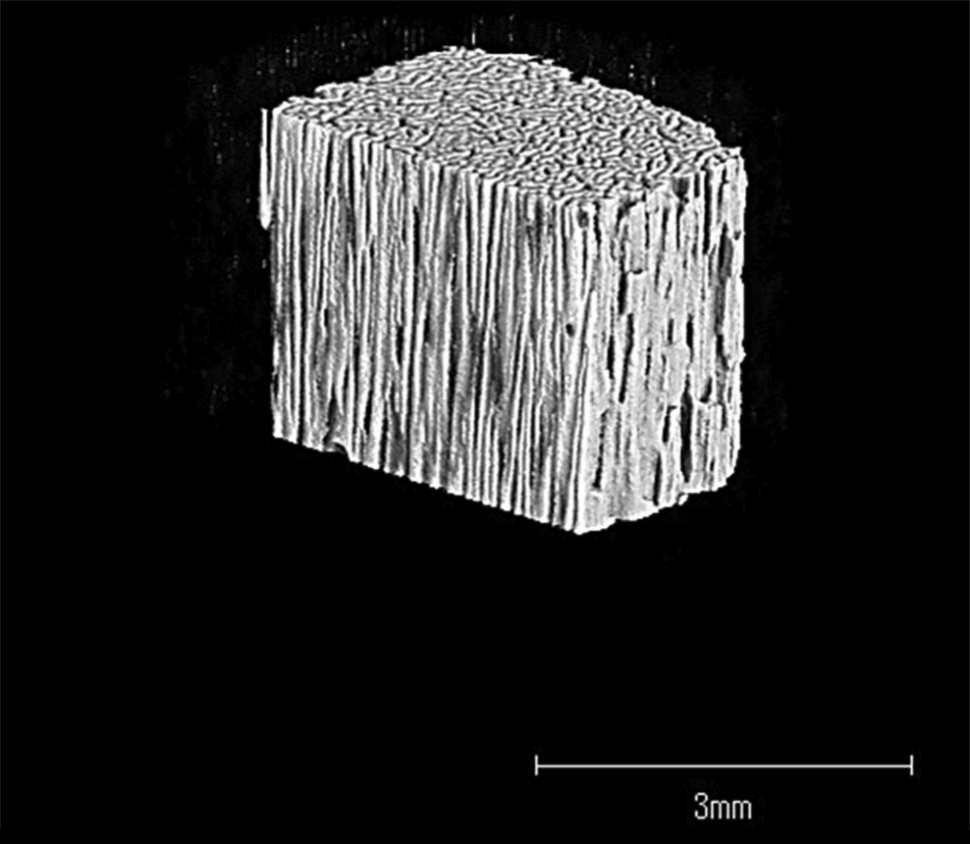
Three-dimensional micro computed tomography image of Affinos^®^, unidirectional porous β-TCP. The pores, 25–300 μm in diameter, are lined up in one direction. The porosity is 57%, and its initial compression strengths of 8 and 1.5 MPa are applied in the directions parallel and perpendicular to the pores, respectively

**Fig. 2:**
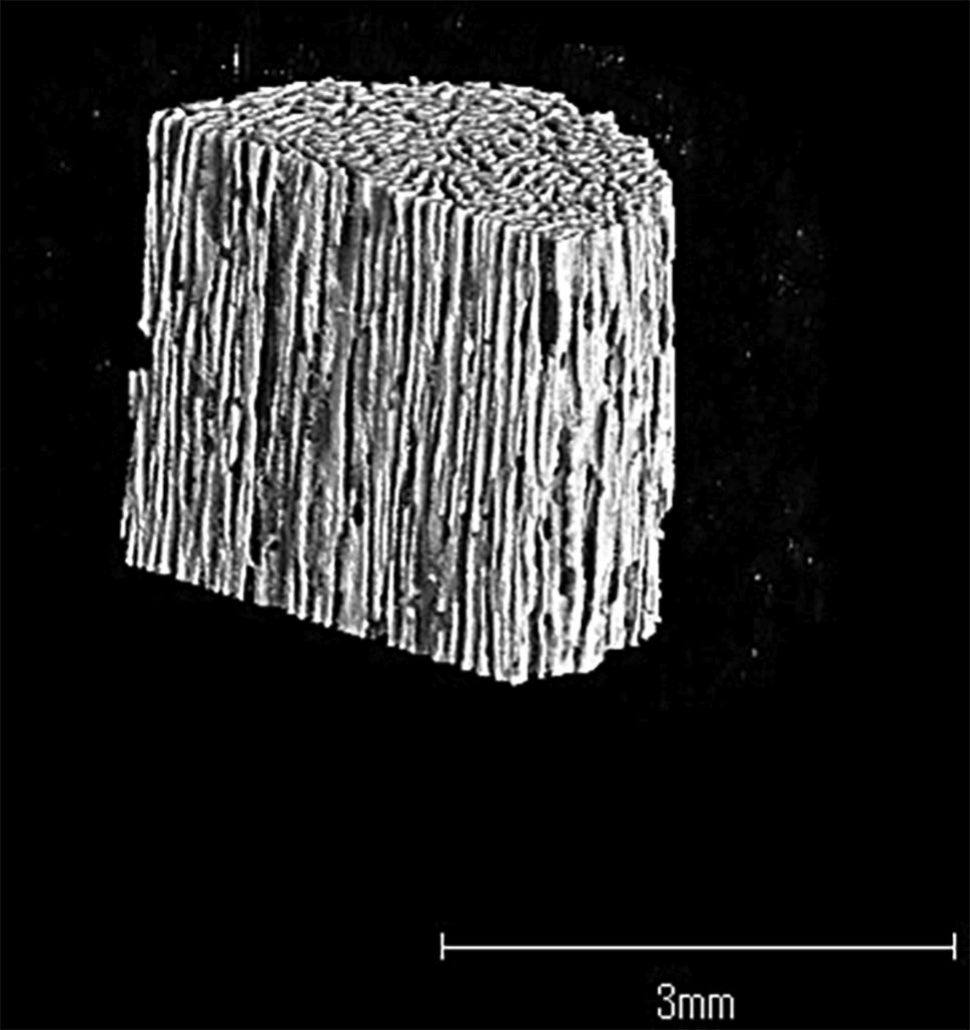
Three-dimensional micro computed tomography image of Regenos^®^, unidirectional porous HAp. The pores, 100–300 μm in diameter, are lined up in one direction. The porosity is 75%, and its initial compression strengths of 14 and 1.0 MPa are applied in directions parallel and perpendicular to the pores, respectively

In collaboration with Kuraray Co., Ltd (Tokyo, Japan), we developed Affinos^®^ and Regenos^®^, artificial bones made of β-TCP and HAp, respectively, that present a unique unidirectional porous structure (Figs. 1, 2). The pores, 25–300 μm in diameter, are lined up in one direction. Artificial bones with a unidirectional porous structure are manufactured by cooling the raw materials with ice in a slurry state, which leads to the formation of ice pillars that are lined up in a vertical direction, like frost columns. When freeze-dried, sublimation of these ice columns produces pores [5]. The greatest feature is that the unidirectional porous structure allows blood to rapidly reach deep inside the material by a capillary effect [6, 7] (Supplementary file1; Affinos^®^, and Supplementary file2; Regenos^®^). Affinos^®^ has a porosity of 57%, and its micropores are believed to promote bone formation [7]. Initial compression strengths of 8 and 1.5 MPa are applied in the directions parallel and perpendicular to the pores, respectively [7]. On the other hand, the porosity of Regenos^®^ is 75%, and initial compression strengths of 14 and 1.0 MPa are applied in directions parallel and perpendicular to the pores, respectively [5].

We herein aimed to comprehensively review the data obtained from animal experiments and the findings in clinical cases, regarding artificial bones with a unidirectional porous structure.

## Animal experiments during the development of Affinos^®^, unidirectional porous β-TCP

From an early stage of development, well-balanced resorption and replacement by the host bone were seen after implantation. This was observed after experimental grafting in the distal femur in a rabbit [8]. In another experiment in which bone grafting for tibial fenestration was performed in rabbits, the material was implanted in touch with both the cortical bone and the medullary cavity. New bone formation was observed throughout the interior of the material at 6 and 12 weeks after implantation. Replacement with host bone was evident in the cortical bone area, while material resorption and remodeling progressed in the medullary cavity [7]. In the same experimental model, histological assessment of the material after injecting dye into the blood vessels 6 weeks after implantation detected the presence of dye along with new bone formation within the material, confirming angiogenesis and the presence of blood flow [9]. Additionally, in an experiment conducted in beagle dogs where grafting was performed in a femoral bony defect, the entire 10-mm-long material was filled with new bone matching the porous direction at 3 months [10]. We examined the optimal mixing ratio of autogenous bone and Affinos^®^ granules in an experimental beagle dog posterolateral lumbar spinal fusion model. It was determined that it is desirable to mix 50% or more of the autogenous bone when using Affinos^®^ for grafting near or outside the bone [11]. In a study from a different facility, it was reported that Affinos^®^ promotes angiogenesis than β-TCP bone with a spherical interconnected porous structure in a vascularized pedicle rat model [12].

## Clinical applications of Affinos^®^, unidirectional porous β-TCP

We have clinically used Affinos^®^ in various orthopedic surgeries and reported those cases. We herein reviewed the published clinical reports in each orthopedic field.

### Trauma surgery

When blocks and granules were used for calcaneal fractures, the artificial bones were resorbed in 3 months and nearly replaced with the patient’s own bone in 6 months [13]. When blocks and granules weighing 25 g, were used for an extensive bony defect during plate fixation in a supracondylar femoral fracture, absorption from the edge of the artificial bone mass was established after 6 months. Observations at 12 and 24 months confirmed that replacement of the material with the patient’s own bone had progressed over time [10]. Although no absorption was seen at 3 months when blocks and granules were used for a large bony defect during plate fixation for pelvic fracture, they were completely replaced with the patient’s own bone at 18 months [14]. Another institution also reported favorable outcomes with the use of Affinos^®^ for bony defects during plate fixation in distal radius fractures [15].

### Osteotomy of the extremities

When blocks and granules were used in the opening wedge during lateral column lengthening for pes planovalgus, resorption was evidenced at 3 months and complete replacement by the patient’s own bone was confirmed at 12 months [10]. When Affinos^®^ blocks and β-TCP with a spherical interconnected porous structure were implanted side by side in the osteotomy site in high tibial osteotomy for knee osteoarthritis, absorption and replacement by the patient’s own bone occurred earlier in Affinos^®^ blocks than in β-TCP with a spherical interconnected porous structure [16, 17].

### Spine surgery

During pedicle screw fixation for fresh vertebral body fractures in older patients, granules were implanted in the bony defect as a vertebroplasty. In cases where loosening of the pedicle screw did not occur, resorption and replacement by the patient’s own bone were observed as early as 3 months after surgery, and in those where loosening did occur due to osteoporosis, delayed onset of resorption was seen, but resorption and replacement by the patient’s own bone were observed 6 months after surgery [18]. In pedicle screw fixation for delayed union of osteoporotic vertebral body fracture in older patients, when granules impregnated with bone marrow blood were implanted into the bony defect of the fractured vertebral body, the defects in the bone cortex in the anterior wall of vertebral body revealed regeneration at 3 months, and the anterior wall fused completely 6 months after surgery [19]. In those cases where blocks were used in the interbody cages during lateral lumbar interbody fusion surgery, the bone fusion rate in the cages was 70.1%. This was equivalent to the results observed when autologous bone blocks are used [20]. During lateral lumbar interbody fusion for intervertebral pseudoarthrosis associated with diffuse idiopathic skeletal hyperostosis, block use within the interbody cages elicited a good bony fusion at 12 months [21]. In the spine, extraosseous bone grafts are frequently used for posterolateral fusion. During posterior occipito-cervical fusion in pediatric patient, when a mixture of autologous iliac cancellous bone and Affinos^®^ granules were implanted between the occipital bone and axis, good bony fusion was observed at 6 months, and remodeling was completed at 12 months [10]. The iliac bone and Affinos^®^ granules were mixed in a one-to-one ratio and transplanted into the pedicle resected during the revision surgery after lumbar long fusion. The pedicle was observed to regenerate by 12 months [10].

### Benign bone tumor surgery

We had reported that when granules were used to fill the lesion after curettage of an enchondroma in the finger bones and intraosseous ganglion, resorption started in 1 month, and the granules were completely replaced with the patient’s own bone by 7–10 months [22]. Similarly, after filling the site of a curetted chondroblastoma in the scapula with 20 g of granules, resorption was clearly seen to start from the margin of the artificial bone at 3 months after surgery, and the complete replacement by the patient’s own bone was observed at 2 years [10].

### Autologous bone harvesting sites

Autologous bone is often harvested in orthopedic surgery. In a long-segment anterior cervical spinal fusion case, a columnar blocks were implanted to fill the site after 7.5 cm of the fibula shaft was harvested [23]. Bone formation occurred from the edge of the fibula at 6 months, and the structure of the cortical bone and medullary cavity, which is originally present in the shaft of a long bone, regenerated in 2 years [10]. We also reported that Affinos^®^ is significantly better than β-TCP of a spherical interconnected porous structure that aids bone regeneration when used to fill harvesting sites of the fibula [24]. When filling the harvest site of the cancellous bone in the distal radius with granules, resorption occurred at 1 month, and the granules were replaced completely with the patient’s own bone by 12 months [10].

## Animal experiments for the development of Regenos^®^; unidirectional porous HAp

During initial development, in an experiment where grafting was performed in the distal femur of a rabbit, new bone formation and angiogenesis were observed inside the material at an early stage [5]. In another rabbit experiment where the graft was inserted into the tibial fenestration in contact with both the cortical bone and the medullary cavity, new bone formation and angiogenesis were observed inside the material early after implantation [25]. The new bone was conserved long-term (2 years after implantation) [26]. In the latter model, an osteon-like structure was observed inside the artificial bone at 26 weeks [6]. The vascular-like structure was preserved inside the artificial bone, and the living bone was retained at 104 weeks [27]. Additionally, in an experiment pertaining to long-term implantation into the tibial fenestration of beagle dogs, it was confirmed that the HAp blocks were partially absorbed and replaced with the host bone after 2 years [28].

In another experiment a beagle high tibial osteotomy model was used as a pre-clinical animal model. New bone formation was observed at the interface between the Regenos^®^ block and the host bone at 6 weeks, indicating excellent osteoconductivity, and bony fusion was achieved in 12 weeks [29]. In a goat laminoplasty model, new bone formation was commonly seen at the laminar spacers implanted between the opened lamina; however, deformation of the spacers was observed to occur at a high rate simultaneously [30].

In an experiment where columnar blocks were implanted in the back muscles of beagle dogs, angiogenesis, and invasion of fibrous tissue were seen inside the artificial bones. However, no new bone formation was observed, clearly indicating that the osteoinductive ability was not similar to that of other HAp materials [31].

Other investigators have reported that a scaffold made of HAp with a unidirectional porous structure was more useful than a spherical interconnected porous structure since it could be impregnated with more cells [32]. When recombinant human bone morphogenetic protein-2 was implanted in bony defects of the skull in mice, significantly more new bone formation was observed than when HAp with a spherical interconnected porous structure was used [33]. Finally, thermal stimulation was found to promote new bone formation in the blocks implanted in rat tibia [34].

## Clinical applications of Regenos^®^; unidirectional porous HAp

We have used Regenos^®^ in various orthopedic surgery. Herein, we reviewed the published cases in each orthopedic field.

### Trauma surgery

In trauma cases, after filling the bony defects with blocks during plate fixation for distal radius fractures, satisfactory new bone formation inside Regenos^®^ block was confirmed by biopsy 12 months later [35]. We had also reported that after filling the bony defects with granules and blocks during internal fixation for calcaneal fractures, bony fusion was achieved at 3 months due to a good osteoconductivity [36].

### Osteotomy of the extremities

We had reported that in high tibial osteotomy for knee osteoarthritis, after we filled the osteotomy site with blocks and granules, adequate bony fusion due to good osteoconductivity and new bone formation were observed at the hinge joints at 6 months. Moreover, some resorption was inclined to occur in blocks at 12 months after implantation [37].

### Spine surgery

Regarding spine surgery, HAp is frequently used as a laminar spacers in cervical laminoplasty. Bone ingrowth has been seen at the interface between the opened lamina and laminar spacers used for double-door laminoplasty, due to their good osteoconductivity. Although deformations (such as cracks, crushing, and wear) and changes in absorption were seen in 21% of cases, the clinical course was not affected [38]. On the other hand, we had reported that Regenos^®^ is not a suitable spacer for open-door laminoplasty since it has been damaged after surgery in many cases, some of which required reoperation [39].

### Benign bone tumor surgery

In a report from another institution, a good bone formation was observed in 44 cases of benign bone tumors in which Regenos^®^ was used as a bone graft after curettage [40]. Moreover, regeneration was evidenced in the cortical bone and resorption and remodeling were observed in the medullary cavity.

### Autologous bone harvesting sites

Also in a report from another institution, when columnar blocks were implanted in the harvesting site of fibula in a pediatric patient, complete regeneration of the host bone with a tubular structure was observed 5 years after surgery [41].

## Discussion

We comprehensively reviewed the data obtained from animal experiments and clinical cases regarding artificial bones with a unidirectional porous structure. The greatest feature is that the unidirectional porous structure allows blood to rapidly reach deep inside the material by a capillary effect [6, 7]. It is reported that pore throat size and connectivity determine bone and tissue ingrowth into porous implants and narrow pore throats inhibited tissue differentiation in pores [42]. In that point, unidirectional porous structure which is unified size has no narrow pore throats. Therefore, tissue could be easily reach deep inside the material through unidirectional pore as well as blood at the shortest distance even though an artificial bone. These are considered as the mechanism of favorable bone regeneration with unidirectional porous structure or bone formation in the unidirectional pore.

In fact, unidirectional porous β-TCP, Affinos^®^ promotes angiogenesis than β-TCP with a spherical interconnected porous structure [12] and absorption and replacement by the patient’s own bone occurred earlier in Affinos^®^ than in β-TCP with a spherical interconnected porous structure [16].

On the other hand, unidirectional porous HAp, Regenos^®^ was also partially absorbed and replaced with the host bone in animal models [27, 28] and clinical cases [37, 40], despite the fact that HAp is basically believed as a non-biodegradable material. Moreover, there is a case report that Regenos^®^ was completely absorbed and regenerated by the host bone in a child [41]. The resorption of interconnected porous HAp implants correlate with the normal bone turnover or activity of osteoclasts in general [43]. The authors speculate that the thin walls of Regenos^®^, by the freeze-casting technique, and the stimulus of blood cells and cytokines from the small vessels may play an important role in the resorption and bone regeneration with bone turnover [28].

In clinical situation, it is difficult to evaluate bone formation or regeneration by histological examination. We reported only one case that satisfactory new bone formation inside the Regenos^®^ was histologically confirmed by biopsy [35]. Therefore, we usually use imaging modality to confirm the new bone formation or replacement with the host bone. Although X-ray is the first choice for these evaluations [44], CT scan is also useful [45]. It is desirable that the evaluation method would be unified in the future.

We are currently conducting research on Affinos^®^, to further expand its original function as a resorbable bone regeneration material. We had previously reported that when teriparatide was administered intermittently after implantation of a block in a rabbit model for tibial fenestration, bone formation inside the artificial bone was significantly promoted when compared to the use of a block implantation alone [46]. Teriparatide is a therapeutic drug for osteoporosis that has a new bone formation effect which is greatly enhanced when used in elderly patients. Experimental results have demonstrated that teriparatide has a good compatibility with Affinos^®^. We are also focusing on the possibilities of platelet-rich plasma (PRP)-impregnated Affinos^®^ by taking advantage of the important ability of artificial bones with a unidirectional porous structure to be rapidly impregnated with blood by a capillary effect. We had reported that both the impregnation amount and rate of PRP with Affinos^®^ were significantly higher than those of β-TCP with a spherical interconnected porous structure [47]. Further clinical applications of Affinos^®^ with teriparatide as well as PRP are expected.

## Conclusion

The unidirectional porous β-TCP, Affinos^®^ has an acceptable performance as a resorbable bone regeneration material. A well-balanced resorption and replacement by the host bone occur early after implantation both in animal and human experiments, including clinical applications. Similarly, animal experiments have confirmed that unidirectional porous HAp, Regenos^®^ is associated with a long-standing vascular-like structure within the pores, and living bone formation. Regenos^®^ has shown excellent osteoconductivity early after implantation in clinical cases. Both of these artificial bones with unidirectional porous structure are suitable for most cases in orthopedic surgery.

## Supplementary Information

Below is the link to the electronic supplementary material.

Supplementary file1 (MPG 3848 KB)

Supplementary file2 (MPG 3196 KB)
